# Chronic Fentanyl Self-Administration Generates a Shift toward Negative Affect in Rats during Drug Use

**DOI:** 10.3390/brainsci11081064

**Published:** 2021-08-13

**Authors:** Angela N. Dao, Nicholas J. Beacher, Vivian Mayr, Annalisa Montemarano, Sam Hammer, Mark O. West

**Affiliations:** 1 Department of Psychology, Rutgers University, Piscataway, NJ 08854, USA; angela.dao@rutgers.edu (A.N.D.); nicholas.beacher@nih.gov (N.J.B.); vvm19@scarletmail.rutgers.edu (V.M.); am2454@scarletmail.rutgers.edu (A.M.); SHammer@epilepsygroup.com (S.H.); 2 Intramural Research Program, National Institute on Drug Abuse, National Institutes of Health, Baltimore, MD 21224, USA

**Keywords:** opioids, fentanyl, self-administration, ultrasonic vocalizations, affect

## Abstract

Drug addiction is thought to be driven by negative reinforcement, and it is thought that a shift from positive affect upon initial exposure to negative affect after chronic exposure to a drug is responsible for maintaining self-administration (SA) in addicted individuals. This can be modeled in rats by analyzing ultrasonic vocalizations (USVs), a type of intraspecies communication indicative of affective state based on the frequency of the emission: calls in the 22 kHz range indicate negative affect, whereas calls in the 50 kHz range indicate positive affect. We employed a voluntary chronic, long-access model of fentanyl SA to analyze affective changes in the response to chronic fentanyl exposure. Male Sprague-Dawley rats self-administered either fentanyl (N = 7) or saline (N = 6) for 30 consecutive days and USVs were recorded at four different time points: the day before the first SA session (PRE), the first day of SA (T01), the last day of SA (T30), and the first day of abstinence (ABS). At T01, the ratio of 50 to 22 kHz calls was similar between the fentanyl and saline groups, but at T30, the ratio differed between groups, with the fentanyl group showing significantly fewer 50 kHz calls and more 22 kHz calls relative to saline animals. These results indicate a shift toward a negative affect during drug use after chronic exposure to fentanyl and support negative reinforcement as a main driving factor of opioid addiction.

## 1. Introduction

Affective state in response to aversive or appetitive stimuli can be readily modeled in laboratory rats by analyzing a form of intraspecies communication known as ultrasonic vocalizations (USVs). USVs can be categorized into two ranges associated with different meanings, due to their emission during emotionally arousing situations: calls in the 18–33 kHz range are referred to as 22 kHz USVs and calls in the 35–70 kHz range are referred to as 50 kHz USVs [1,2].

Due to their predictable occurrence in response to aversive situations, 22 kHz calls are considered representative of a negative affective state and are generated during common behavioral situations such as exposure to predators [3], unfamiliar touch [4], drug withdrawal [5,6], and exposure to pain such as foot shock [7]. Conversely, 50 kHz calls are reliably emitted during situations involving the anticipation of potential reward [8,9], social contact [10], electrostimulation of mesolimbic sites supporting self-stimulation [11], and the administration of addictive drugs [12,13]. Therefore, 50 kHz calls are representative of an appetitive, hedonic behavioral state associated with positive affect [9,10]. As both 22 and 50 kHz USVs can provide insight into the affective state of animals, they can be recorded and analyzed during periods of drug administration to identify changes in affect associated with chronic opioid exposure.

It is commonly accepted that negative reinforcement is the driving factor behind addiction [14], whereby the escape or avoidance of negative affect during drug use and withdrawal is the main motivating factor maintaining the self-administration of addictive drugs [15]. Furthermore, a shift from positive affect upon initial drug use to the emergence of negative affect after extended drug exposure and development of drug dependence and/or addiction is a salient motivational factor in chronic, long-access animal models [16] and human drug abuse [17]. Analyses of rodent USVs during opioid administration indicated that anticipatory 50 kHz emissions decrease after repeated exposure to morphine, suggesting an aversive influence of morphine exposure [12] and supporting a shift from positive to negative affect in response to repeated opioid administration.

Morphine was shown to dose-dependently suppress both 22 and 50 kHz USVs independent of opioid effects on pain perception, and this response can be attenuated by administration of a mu-opiate receptor antagonist such as naloxone [18], implicating opioid specificity. Additionally, opioid administration suppresses 22 kHz USVs emitted during foot shock, whereas the audible pain-associated squeak is unaffected, further dissociating USV suppression by opioids from their analgesic effects [7]. These results are consistent with claims that opioids influence affective as well as autonomic, somatic, and motor processes, and indicate a role of opioids in modifying the central mechanisms of USVs.

Surprisingly, little emphasis has been placed on understanding the specific affective state of rats during the self-administration of opioids. Therefore, we employed a long-access fentanyl self-administration (SA) model to analyze the changes in affect in response to chronic fentanyl exposure in rats. Intravenous SA is the most translatable animal model for human drug addiction and is thus often referred to as the gold standard for measuring abuse liability [19,20]. The model establishes two key aspects of drug addiction: compulsive drug use and escalation of drug intake over time. The inclusion of USV analysis allows for the identification of the emergence of a negative affective state, which is a third key aspect of drug addiction. Based on previous studies citing a shift from positive to negative affect after repeated drug exposure [12,16,17], we predicted that rats would exhibit increased positive affect upon initial exposure to fentanyl, as indicated by increased 50 kHz call rates during the first session of fentanyl SA, which would then shift toward negative affect after 30 days of fentanyl SA.

## 2. Materials and Methods

### 2.1. Animals

Adult, male Sprague-Dawley rats (Charles River, Wilmington, MA, USA) were allowed to self-administer the opiate receptor agonist fentanyl HCL (dose = 2.57 μg/kg per i.v. infusion; fentanyl SA group, N = 7) or saline (saline SA group, N = 6) for 30 consecutive days. Rats were singly housed on a 12:12 h light:dark cycle (lights on at 10:30 a.m.). Prior to surgery, rats were allowed to reach adult weight (350 g) and maintained at this weight thereafter to avoid the addition of fat tissue.

### 2.2. Catheterization Surgery

Animals were anesthetized with a ketamine/xylazine (K/Xyl) mixture (50 mg/kg, i.p.) and given an injection of atropine (10 mg/kg; i.p.) to decrease fluid buildup in lungs and prevent respiratory arrest. Anesthesia was monitored and maintained throughout surgery by intermittent K/Xyl injections. During surgery, animals were chronically implanted with an intravenous catheter in the right jugular vein. This catheter was threaded subcutaneously and exited at the scalp where it was led through a j-shaped stainless-steel cannula attached to the skull using dental cement and jeweler’s screws. The catheter was protected by a metal spring-leash permanently connected to the animal’s cannula to prevent damage. Following surgery, the animal was housed in a self-administration operant chamber at all times for the entirety of the SA experiment. Animals were allowed one week to recover from surgery, during which time they received once daily i.v. infusions of antibiotics and NSAID pain reliever (rimadyl and baytril). During all hours other than SA sessions, a 200 µL infusion of saline was delivered every 25 min by a computer-controlled syringe pump to preserve catheter patency. Animals received water ad libitum and received enough food to maintain a weight of 350 g throughout the duration of the experiment.

### 2.3. Self-Administration Apparatus

The clear Plexiglas chamber in which animals were housed included a corner with a fixed 6-photocell device used to monitor and record head movements [21]. An infusion was administered only when a correct operant response was performed in this corner. A correct operant response consisted of breaking photocells 2 and 3 in succession within 1 s. All rewarded responses (RRs) and unrewarded responses (URs) were recorded. The Plexiglas chamber was housed within a ventilated, sound-attenuating outer shell.

### 2.4. Self-Administration

SA sessions (6 h/day, 7 days/week) were conducted using the long-access model of Ahmed and Koob [22], which models human addiction, including escalation of intake and persistent increase in the motivation for drug-taking [23,24]. SA sessions ran each day for 30 consecutive days, starting at light onset. Sessions automatically ended upon the completion of 6 h. During the session, drug or saline was available during the entire 6 h on an FR1 reinforcement schedule. A correct response (except during timeout, see below) turned on the pump and automatically dispensed a 0.9 μg/0.075 mL solution of intravenous fentanyl (or an equal volume of saline) through the surgically implanted catheter over 2.5 s, for an average infusion dose of 2.57 μg/kg. This correct response was defined as an RR. An RR immediately triggered a 40 s inter-trial interval timeout as a precautionary measure to prevent overdose, but all responses provided during this time were recorded as URs.

### 2.5. USV Recording and Scoring

Prior to the commencement of SA recording sessions, a condenser microphone (CM16/CMPA, Avisoft) was suspended 2.5 cm above an arrangement of small holes in the top of the Plexiglas SA chamber. USVs were recorded at a 250 kHz sampling frequency (16 bits) using recording software (Ultrasound Gate, Avisoft, Glienicke/Nordbahn, Germany). Baseline USVs were recorded one week after surgery prior to the start of the first SA session over the same 6 h period as SA sessions were conducted. Subsequent USV recordings were obtained for 6 h during session 1 (T01), session 30 (T30), and the first day of abstinence (ABS; session 31) at the same time of day as a 6 h SA session. As characterization and scoring of USVs are time- and labor-intensive, this limited agenda was designed to capture affective calling during SA for the first time, SA for the 30th consecutive day, and the first time being deprived of the expected drug (18 h withdrawal).

Audio files were run through an automated detector, DeepSqueak [25], to isolate potential calls. These were then manually checked to distinguish between actual calls (which were accepted) and artifacts and background noise (which were rejected). The automatic detector outputs the specific frequency and exact timing of individual calls. Only manually accepted calls were used for analyses. Calls were designated as belonging to the 22 or 50 kHz ranges.

### 2.6. Data Analyses

Data were analyzed using Prism GraphPad software. Behavioral measures included (i) number of RRs/session, (ii) average drug level (mg/kg) maintained during SA, (iii) slope of escalation of intake, and (iv) 22 and 50 kHz call rates during baseline, T01, T30, and ABS sessions.

#### 2.6.1. Escalation of Intake

The total number of RRs was regressed over sessions 1 through 30 for both fentanyl and saline SA. The analysis was conducted using a simple linear regression, where RR was defined as the dependent variable and session was defined as a continuous independent variable. An additional linear regression was performed in which total fentanyl intake was regressed over the session (1–30), where intake was defined as a dependent variable and session was defined as a continuous independent variable. To incorporate body weight into the calculation of total fentanyl intake, the following equation was used: intake = (#RRs × μg fentanyl per infusion)/body weight.

#### 2.6.2. USV Analysis

One-tailed *t*-tests were conducted to compare the ratio of 50 kHz to 22 kHz USVs for T01 vs. T30. One-tailed t-tests were performed based on expectations derived from the literature, from which we formed the hypothesis that the ratio would shift toward fewer positive and more negative calls at T30 relative to T01.

## 3. Results

### 3.1. Acquisition of Fentanyl SA

**Figure 1:**
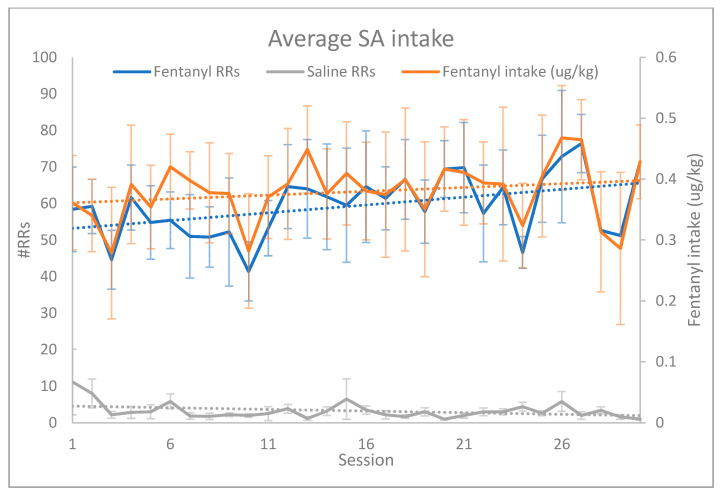
Average SA intake. A simple linear regression revealed that fentanyl animals exhibited escalation of intake over 30 days of SA training as demonstrated by the increased number of fentanyl infusions (RRs; F(1,28) = 16.45, *p* = 0.0004) and average daily intake of fentanyl (accounting for body weight; μg/kg; F(1,28) = 9.912, *p* = 0.0039). Saline animals did not escalate intake over 30 days of SA (F(1,28) = 4.140, *p* > 0.05). Error bars denote the SEM.

A total of 28 rats were surgically prepared for SA. Seven rats in the fentanyl SA group, and six rats in the saline SA group completed all phases of SA and USV recording. Animals self-administered fentanyl in a manner consistent with animal models of substance use disorder, in which escalation of intake is a key marker of addiction [26]. The average number of reinforced responses (#RRs) was plotted against SA session (session 1–30). The simple linear regression revealed that the slope of the line for fentanyl SA (0.6242) was significantly different from zero (F(1,28) = 16.45, *p* = 0.0004), identifying escalation of intake over time. Accounting for body weight, a separate linear regression similarly identified escalation of fentanyl intake (µg/kg) over time (F(1,28) = 9.912, *p* = 0.0039, Figure 1). The average number of RRs in the saline group remained low and did not change across sessions (Figure 1; F(1,28) = 4.140, *p* > 0.05).

### 3.2. Shift toward Negative Affect after 30 Days of Fentanyl SA

**Figure 2:**
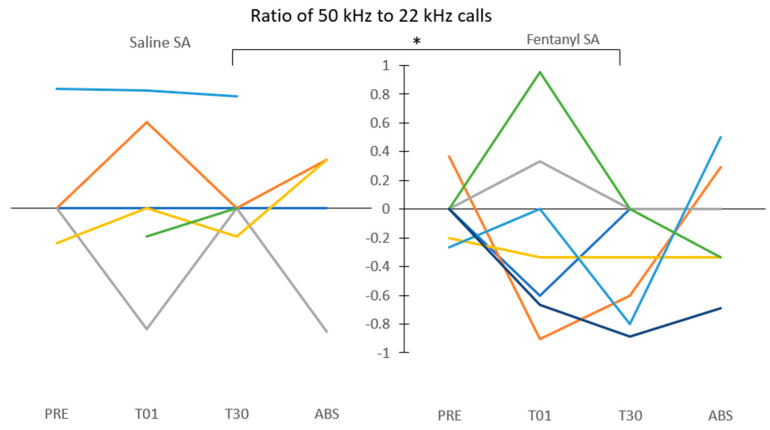
Ratio of 50 to 22 kHz calls. Tracking of all rats’ 50 kHz vs. 22 kHz calls across all sessions. Each line represents one rat. Y-axis = (B − A)/(B + A), where B is the rate of 50 kHz USVs and A is the rate of 22 kHz USVs. Thus, numbers above the horizontal line at zero represent a higher ratio of positive to negative calls; numbers below zero indicate a higher ratio of negative to positive calls; zero (horizontal line) indicates equal rates of the two calls. Note the absence of positive USVs and the prevalence of negative USVs in the 30th fentanyl SA session. The ratio did not differ between groups on T01, but the negative ratio of the fentanyl group was significantly different from the ratio of the saline group at T30; * *p* = 0.02.

**Figure 3:**
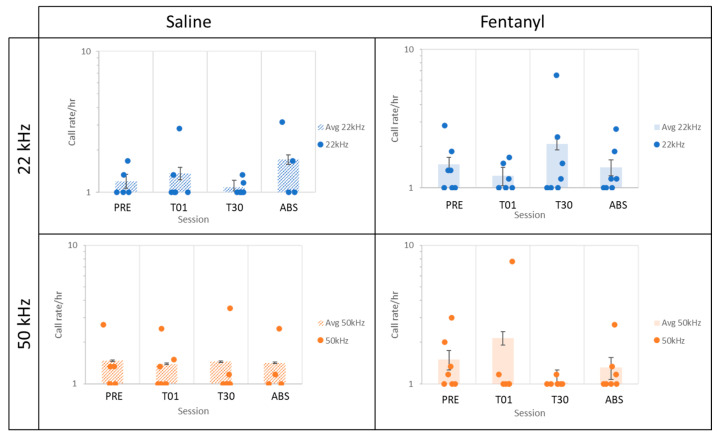
The 22 kHz and 50 kHz call rates per hour. Each bar represents the mean call rate per hour at PRE, T01, T30, and ABS for both saline and fentanyl groups. Each dot represents one animal. The fentanyl group exhibited a shift toward negative affect after 30 days of fentanyl SA as indicated by the increase in 22 kHz calls and decrease in 50 kHz calls at T30 compared to T01. This change was not observed in the saline group. Two data points (one fentanyl T01 and one saline PRE) were outliers and removed from Figure 3*,* but not from statistical analyses, to avoid distortion of the y-axis, so that the visualization of the data is consistent with the results showing a shift toward negative affect at T30. Note the non-linear scale on the y-axis. Error bars denote the SEM.

Animals exhibited a mix of affective responses to fentanyl SA in session 1 (T01). Relative to baseline (PRE), some rats emitted more 50 kHz than 22 kHz calls during the six hours of T01, whereas others exhibited the opposite pattern (Figure 2). However, by session 30 (T30), no fentanyl SA rats emitted more 50 kHz than 22 kHz calls, with several rats emitting more 22 kHz than 50 kHz calls. Across all sessions, the average 50 kHz call rate per hour peaked at T01 but declined to near zero at T30 (Figure 3). Combined, the seven rats in the fentanyl group emitted a total of one 50 kHz call during the six hours of T30. In contrast, the average 22 kHz call rates peaked at T30.

To test the hypothesis that affective state would become more negative after a month of daily fentanyl SA, a key planned comparison was between the ratio of 50 to 22 kHz call rates at T01 vs. T30. The ratio was computed for each animal in each session using the formula (B − A)/(B + A), where B is the 50 kHz call rate and A is the 22 kHz call rate. Using this formula, positive values indicate a higher prevalence of 50 kHz calls, negative values indicate a higher prevalence of 22 kHz calls, and zero indicates equal rates of each (Figure 2). At T01, the fentanyl group (M = −0.17, SD = 0.65) showed no difference from the saline group (M = 0.06, SD = 0.59) (t(11) = −0.684, *p* = 0.25). In contrast, at T30, the fentanyl group (M = −0.37, SD = 0.39) showed a significant shift toward fewer 50 kHz and more 22 kHz calls compared to the saline group (M = 0.10, SD = 0.35) (t(11) = −2.31, *p* = 0.02). Thus, the ratio of 50 kHz to 22 kHz calls was not different between groups at T01 but shifted to a significantly more negative value in the fentanyl group at T30 (Figure 2). These results indicate the emergence of a predominately negative affective state during a six-hour session of drug use after a month of chronic fentanyl self-administration.

## 4. Discussions

The present results provide evidence of a shift toward negative affect during chronic opioid administration, supporting negative reinforcement as a salient motivating factor driving drug addiction. The decrease in 50 kHz and increase in 22 kHz USVs in session 30 relative to session 1 of fentanyl but not saline SA suggest an aversive response to repeated opioid use, which is consistent with previous reports [12]. The call rate/hour results (Figure 3) are reinforced by the data presented by the ratio of 50 kHz calls to 22 kHz calls (Figure 2), which track animals across sessions and highlight individual differences among animals. This ratio provided a useful measure of general affect, which varied between positive and negative during the long-access sessions in the present study. T30 in the fentanyl group was the only recording session exhibiting an equal or greater ratio of negative to positive calls for all rats, as well as a dearth of 50 kHz calls, indicating not only a lack of positive affect but also a shift toward overall negative affect. Accordingly, these results further corroborate those of previous studies reporting a shift from positive to negative affect after chronic drug abuse [12,16,17].

We observed no significant or uniform increase in positive affect at T01 of fentanyl SA, consistent with previous studies reporting the suppression of 50 kHz calls in response to opioids in drug-naïve rats [27,28]. The initial exposure to opioids does not consistently generate a positive affective response [29,30] (for a review, see Verendeev and Riley [31]) typically observed with other classes of drugs [16,32]. Given the presence of some negative affective responses to fentanyl SA on T01, we cannot rule out the possibility that the increase in 22 kHz calls at T30 could have been associated with a higher drug intake in that session. That increase in negative calls plus the lack of major changes in the call rates of either frequency at ABS highlight the presence of negative affect during drug use after repeated exposure to the drug, not just once the drug has left the body and withdrawal sets in. The mix of both 22 and 50 kHz calls during the 6 h ABS session may reflect fluctuations between a negative affective state associated with opioid withdrawal [6] and a positive affective state associated with anticipation of drug for the 31st consecutive day, since 50 kHz calls can indicate an anticipatory state [8,33,34].

Opioids have been known to suppress USVs in general [12,18,28]. This appears to be the case in the present study, considering the low call rates detected compared to the higher rates of both 22 and 50 kHz calls detected during cocaine SA (e.g., [16,35]). The pattern of USV emission during fentanyl SA appears to be notably different from the SA of other classes of drugs, such as stimulants. Behaviorally, we did not observe an initial “load-up” period in which drug level rises rapidly at the beginning of each SA session as is observed with cocaine SA [36]. Cocaine drug level is a strong predictor of affect, such that the 50 kHz emissions by animals self-administering cocaine coincide with rising drug levels exclusively during initial load-up, coinciding with a decrease in the 22 kHz call rate. Thereafter, 22 kHz calls dominate the maintenance phase, increasing whenever drug level falls [37]. Cocaine generates a positive affect upon first exposure to SA [16], along with motoric activation and emotional arousal, which is the driving force for the emission of USVs [8]. This does not appear to be the case for fentanyl SA, as initial exposure to fentanyl SA generated neither positive nor negative affect in the present study. Opioid SA is not as clearly associated with emotional arousal as that of cocaine, considering environmental preferences with respect to each drug. Badiani et al. [19] reviewed extensive evidence that both rats and humans prefer recreational and/or social use of the psychomotor stimulant cocaine, whereas they prefer taking opioids in private settings. Fentanyl SA may be associated with greater tranquility, less emotional arousal, and less tendency to emit positive vocalizations. 

We observed a trend toward elevated 50 kHz call rates during the first SA session, similar to that of Avvisati et al. [38], who also studied opioid SA in rats occupying their home cage. They reported a further increase in 50 kHz calls during the first 30 min of SA sessions after two weeks of heroin SA. That finding, however, differs from the decline we observed in the 50 kHz call rate after a month of daily fentanyl SA. A second difference between the studies is the paucity of 22 kHz calls in their study compared to the present increase in 22 kHz calls during session 30 of fentanyl SA. These differences may relate to the SA of the two different opioids, their use of 3 h sessions for 14 days vs. the 6 h session model of addiction [22] for 30 days used in the present study, or their daily alternation with cocaine SA. Resolving these differences will be important for understanding affective processes in opioid abuse.

Some insight may be gained from our prior studies of cocaine SA. During load-up, the spike in 50 kHz call rates increased [16] or was sustained [37] across 14 sessions of cocaine SA, similar to the increase across sessions observed by Avvisati et al. [38] during the first 30 min of their sessions. Following load-up on cocaine, the 5+ hour maintenance phase was devoid of 50 kHz calls but dominated by 22 kHz calls, indicating that responding was being maintained by negative reinforcement [37]. A similar predominance of 22 kHz calls indicating negative affect and potentially negative reinforcement may have characterized the maintenance of fentanyl SA. However, we observed substantial variability within and between subjects in the timing of fentanyl self-infusions, with no clear separation of load-up from maintenance. Therefore, we chose not to attempt analyses of the initial portion of sessions, but instead analyzed the whole session.

The present decline in positive affect in parallel with an increase in negative affect across chronic opioid SA is consistent with the decrease to below baseline in subjective “liking” of the effects of fentanyl administration in human subjects over time [39,40], in agreement with the incentive sensitization hypothesis of Berridge and Robinson [39]. Our findings in self-administering rats indicate that repeated exposure to fentanyl generates a significant shift toward negative affect even while on the drug. This suggests that chronic fentanyl SA involves aversive effects, despite outward signs that might appear consistent with models of addiction emphasizing positive reinforcement. Multiple ascending pathways originating in the brainstem are responsible for the generation of emotional arousal, including the ventral dopaminergic system originating in the ventral tegmental area (VTA) [8], a pathway strongly implicated in the rewarding effects of both opioids and stimulants. In the case of stimulants such as cocaine, the lateral habenula has been implicated in playing a critical role in the regulation of negatively motivated behaviors by targeting midbrain neuromodulatory systems such as the dopaminergic pathway projecting from the VTA [41,42]. Negative behaviors associated with opioids, however, are predominantly modulated by the paraventricular nucleus of the thalamus, which has significant inputs to the nucleus accumbens, whereby it mediates the negative signs of withdrawal and opioid-related aversive memory [43]. Therefore, it is possible that differences in affective regulation during cocaine and versus SA may be a result of activity in separate neural circuits during exposure to different classes of drugs.

We previously reported that the only USVs emitted during the maintenance phase of cocaine SA are 22 kHz calls [37]. Those negative calls were associated with falling cocaine levels, suggesting that further study is necessary of the relationship between USVs and fluctuating levels of fentanyl in the blood. Whereas the pattern of fentanyl SA and its associated affect are markedly different from those of cocaine SA, our findings with both fentanyl and cocaine [16] are consistent with the literature, supporting negative reinforcement as a driving factor of addiction and emphasizing that escape/avoidance of negative affect is a salient motivational factor reinforcing drug addiction.

## 5. Conclusions

Chronic fentanyl SA generates a shift toward negative affect during drug use as indicated by an increase in 22 kHz calls and a decrease in 50 kHz calls at T30 compared to saline control animals. The shift toward a negative affect during drug use is consistent with previous reports of increased negative affect in response to chronic drug exposure [12,16,17] and supports negative reinforcement as a salient motivational factor driving drug addiction.

## Data Availability

The data presented in this study are available on request from the corresponding author.
